# HB-EGF upregulates StAR expression and stimulates progesterone production through ERK1/2 signaling in human granulosa-lutein cells

**DOI:** 10.1186/s12964-022-00983-4

**Published:** 2022-10-25

**Authors:** Jung-Chien Cheng, Xiaoyu Han, Qingxue Meng, Yanjie Guo, Boqun Liu, Tinglin Song, Yuanyuan Jia, Lanlan Fang, Ying-Pu Sun

**Affiliations:** Center for Reproductive Medicine, Henan Key Laboratory of Reproduction and Genetics, The First Affiliated Hospital of Zhengzhou University, 40, Daxue Road, Zhengzhou, Henan China

**Keywords:** HB-EGF, Granulosa cells, Steroidogenesis, StAR, Progesterone

## Abstract

**Background:**

Heparin-binding epidermal growth factor-like growth factor (HB-EGF) belongs to the epidermal growth factor (EGF) family of growth factors. HB-EGF and its receptors, epidermal growth factor receptor (EGFR) and HER4, are expressed in the human corpus luteum. HB-EGF has been shown to regulate luteal function by preventing cell apoptosis. Steroidogenesis is the primary function of the human corpus luteum. Steroidogenic acute regulatory protein (StAR) plays a critical role in steroidogenesis. StAR expression and progesterone (P4) production in human granulosa-lutein (hGL) cells have been shown to be upregulated by a ligand of EGFR, amphiregulin. However, whether HB-EGF can achieve the same effects remains unknown.

**Methods:**

A steroidogenic human ovarian granulosa-like tumor cell line, KGN, and primary culture of hGL cells obtained from patients undergoing in vitro fertilization treatment were used as experimental models. The underlying molecular mechanisms mediating the effects of HB-EGF on StAR expression and P4 production were explored by a series of in vitro experiments.

**Results:**

Western blot showed that EGFR, HER2, and HER4 were expressed in both KGN and hGL cells. Treatment with HB-EGF for 24 h induced StAR expression but did not affect the expression of steroidogenesis-related enzymes, P450 side chain cleavage enzyme, 3β-hydroxysteroid dehydrogenase, and aromatase. Using pharmacological inhibitors and a siRNA-mediated knockdown approach, we showed that EGFR, HER4, but not HER2, were required for HB-EGF-stimulated StAR expression and P4 production. In addition, HB-EGF-induced upregulations of StAR expression and P4 production were mediated by the activation of the ERK1/2 signaling pathway.

**Conclusion:**

This study increases the understanding of the physiological role of HB-EGF in human luteal functions.

**Video Abstract**

**Supplementary Information:**

The online version contains supplementary material available at 10.1186/s12964-022-00983-4.

## Background

Heparin-binding epidermal growth factor-like growth factor (HB-EGF) is a member of the epidermal growth factor (EGF) family of growth factors that consists of EGF, transforming growth factor-α, amphiregulin, betacellulin, epiregulin, and neuregulins [1]. HB-EGF was initially identified as a 22 kDa secreted protein and a potent mitogen for smooth muscle cells from the macrophage-like U937 cell-conditioned medium by heparin-affinity chromatography [2]. HB-EGF is first synthesized as a transmembrane protein, pro-HB-EGF, which is then cleaved to yield the soluble mature form of HB-EGF through a regulated proteolytic process known as ectodomain shedding [3]. Like other members of the EGF family, the biological functions of HB-EGF are mediated through interaction with the ErbB receptor family. It is known that HB-EGF binds EGFR/ErbB1 and HER4/ErbB4 to regulate different cellular functions [4]. HB-EGF has been shown to regulate various physiological processes and participate in many pathological events [5–7]. HB-EGF is involved in the regulation of reproductive function. However, most studies focused on its expression in the uterus and its role in regulating blastocyst adhesion, growth, and differentiation [5, 8].

Steroidogenesis is one of the primary functions of the ovary. Progesterone (P4) production by the corpus luteum is essential for establishing normal pregnancy until the placenta becomes competent to take over the role of the P4 production. Luteal phase deficiency is one of the major causes of female infertility and a clinical diagnosis associated with inadequate P4 production [9]. Administration of pregnant mare serum gonadotropin (PMSG)-primed mouse ovaries with hCG reduces *Hb-Egf* first, but its expression is restored and increased during luteinization, which suggests that HB-EGF plays a potential role in the corpus luteum [10]. *Lgr4* deficiency (*Lgr4*^*-/-*^) mice exhibit defects in P4 synthesis and luteinization. Exogenous treatment of HB-EGF rescues the luteinization deficiency of granulosa cells in *Lgr4*^*-/-*^ mice [10]. In humans, the expression of HB-EGF and its receptors, EGFR and HER4, are detected in the corpus luteum [11]. Treatment of recombinant human HB-EGF inhibits apoptosis in human granulosa-lutein (hGL) cells [12]. These results provide evidence that HB-EGF may play a critical role in the regulation of luteal growth.

Steroidogenic acute regulatory protein (StAR)-mediated transportation of the cholesterol from the outer to the inner membrane of the mitochondria is the key regulatory step in ovarian steroidogenesis [13, 14]. It is well characterized that ovarian StAR expression is primarily regulated by follicle-stimulating hormone (FSH) and luteinizing hormone (LH) through the cAMP-PKA signaling pathway [15]. However, increasing evidence has demonstrated that ovarian StAR expression is also regulated by many local factors via an autocrine or paracrine fashion. Treatment of mouse cumulus-oocyte complexes with amphiregulin (AREG), an EGFR ligand, stimulates *Star* expression [16]. Our previous study shows AREG upregulates StAR expression and P4 production in hGL cells. The stimulatory effects of AREG on StAR expression and P4 production require the activation of the ERK1/2 signaling pathway [17]. To date, the effects of HB-EGF on StAR expression and P4 production in hGL cells are not known. The KGN cell line is a human granulosa-like tumor cell line derived from a 63-year-old Japanese woman. Unlike other established human granulosa cell lines, KGN cells express functional FSH receptor and retain many normal physiological characteristics of granulosa cells, including steroidogenesis [18]. To make the experiments more technically feasible, especially for those gene silencing experiments, we used KGN cell line as our major in vitro model together with primary hGL cells obtained from patients undergoing in vitro fertilization (IVF) treatment to investigate the effects and related underlying molecular mechanisms of HB-EGF on StAR expression and P4 production in human granulosa cells.

## Methods

### Antibodies and reagents

The p-EGFR (#3777), p-HER2 (#2243), HER2 (#2165), p-HER4 (#4757), HER4 (#4795), p-ERK1/2 (#9106), ERK1/2 (#9102), phospho-AKT (#9271), and AKT (#9272) antibodies were purchased from Cell Signaling Technology. The EGFR (#sc-373746) and α-tubulin (#sc-23948) antibodies were purchased from Santa Cruz Biotechnology. The recombinant human HB-EGF was obtained from R&D systems. The AG1478, AG825, and LY294002 were obtained from Sigma. The U0126 was obtained from Cayman.

### Cell culture and treatments

The human granulosa cell tumor-derived cell line, KGN [18], was kindly provided by Professor Aaron Hsueh in the Department of Obstetrics and Gynecology at Stanford University. The primary human granulosa-lutein (hGL) cells were purified by density centrifugation from follicular aspirates collected from women undergoing oocyte retrieval, as previously described [19]. Cells were cultured in a humidified atmosphere containing 5% CO_2_ and 95% air at 37 °C in Dulbecco’s Modified Eagle Medium/nutrient mixture F-12 Ham medium (DMEM/F-12; Gibco) supplemented with 10% charcoal/dextran-treated FBS (HyClone), 100 U/mL of penicillin and 100 µg/mL of streptomycin sulfate (Boster). For HB-EGF treatments, KGN or primary hGL cells were cultured in 6-well plates with 2 mL of culture medium. Cells were serum-starved in a medium without FBS for 24 h to induce quiescence before treatments. All treatments for cells were performed in a medium without FBS. The recombinant human HB-EGF was solubilized in phosphate-buffered saline (PBS). The AG1478, AG825, U0126, and LY294002 were dissolved in dimethyl sulfoxide (DMSO). For the experiments using a pharmacological inhibitor, cells were pretreated with 10 µM AG1478, 10 µM AG825, 10 µM U0126, or 10 µM LY294002 for 1 h in a medium without FBS. After 1 h incubation with inhibitor, HB-EGF was added directly into the culture well. All groups in each experiment were exposed to all the relevant vehicles for that experiment. For KGN cells, the individual experiment was repeated at least three times by using different passages of cells. Individual primary cultures were composed of cells from one individual patient. For primary hGL cells, each experiment was repeated at least three times and each time used cells derived from different patients.

### Reverse transcription quantitative real-time PCR (RT-qPCR)

Total RNA was extracted with the TRIzol (Invitrogen) according to the manufacturer’s instructions. RNA (1 µg) was reverse-transcribed into first-strand cDNA with the iScript Reverse Transcription Kit (Bio-Rad Laboratories). Each 20 µL qPCR reaction contained 1X SYBR Green PCR Master Mix (Applied Biosystems), 60 ng of cDNA, and 250 nM of each specific primer. The primers used were 5′-CAG GAG GGG TGG ACA CGA C-3′ (sense) and 5′-AGG TTG CGT GCC ATC TCA TAC-3′ (antisense) for P450 side chain cleavage enzyme (*CYP11A1*); 5′-GCC TTC CAG ACC AGA ATT GAG AGA-3′ (sense) and 5′-TCC TTC AAG TAC AGT CAG CTT GGT-3′ (antisense) for 3β-hydroxysteroid dehydrogenase type II (*HSD3B2*); 5′-AAA CTT ACG TGG CTA CTC AGC ATC-3′ (sense) and 5′-GAC CTG GTT GAT GCT CTT G-3′ (antisense) for steroidogenic acute regulatory protein (*StAR*); 5′-GAG AAT TCA TGC GAG TCT GGA-3′ (sense) and 5′-CAT TAT GTG GAA CAT ACT TGA GGA CT-3′ (antisense) for *CYP19A1* aromatase; 5′-GGT GCA GGA GAG GAG AAC TGC-3′ (sense) and 5′-GGT GGC ACC AAA GCT GTA TT-3′ (antisense) for *EGFR*; 5′-CAG CCT CCC ATC TGC ACT AT-3′ (sense) and 5′-GCC ATC CTT GAA AAC TCA GC-3′ (antisense) for *HER4*; and 5′-GAG TCA ACG GAT TTG GTC GT-3′ (sense) and 5′-GAC AAG CTT CCC GTT CTC AG-3′ (antisense) for *GAPDH*. The qPCR was performed on an Applied Biosystems QuantStudio 12 K Flex system equipped with 96-well optical reaction plates. The specificity of each assay was validated by melting curve analysis and agarose gel electrophoresis of the PCR products. All of the RT-qPCR experiments were run in triplicate, and a mean value was used to determine the mRNA levels. RNAase-free water and mRNA without RT were used as negative controls. Relative quantification of the mRNA levels was performed using the comparative Ct method with GAPDH as the reference gene and using the formula 2^−∆∆Ct^.

### Western blot analysis

Cells were lysed in cell lysis buffer (Cell Signaling Technology) supplemented with a protease inhibitor cocktail (Sigma). The protein concentration was measured by the BCA protein assay kit (Thermo Scientific). Samples with an equal amount of protein were separated by SDS-polyacrylamide gel electrophoresis and transferred to PVDF membranes. After 1 h blocking at room temperature with 5% non-fat dry milk in Tris-buffered saline (TBS), the membranes were incubated overnight at 4 °C with primary antibodies diluted in 5% non-fat milk/TBS. Following primary antibody incubation, the membranes were washed with TBS and subsequently incubated with appropriate HRP-conjugated secondary antibodies (Bio-Rad Laboratories). The immunoreactive bands were detected with an enhanced chemiluminescence kit and imaged with a ChemiDoc MP Imager (Bio-Rad Laboratories).

### Small interfering RNA (siRNA) transfection

To knock down endogenous EGFR or HER4, KGN cells were cultured in 6-well plates and grown to 70–80% confluence. Cells were transfected with 50 nM ON-TARGETplus SMARTpool siRNA targeting a specific gene (Dharmacon) using Lipofectamine RNAiMAX (Invitrogen) as per the manufacturer’s instructions in a culture medium supplemented with 10% FBS for 24 h. On the next day, cells were serum-starved in a medium without FBS for 24 h to induce quiescence before treatments. The 50 nM ON-TARGETplus siCONTROL NON-TARGETING pool siRNA was used as the transfection control. The knockdown efficiency of specific siRNA was examined by RT-qPCR or western blot.

### ELISA assay for progesterone

Progesterone (P4) levels were measured using an ELISA Kit (#582,601, Cayman) as per the manufacturer’s instructions. The interassay CV and intraassay CV for P4 ELISA were < 10%. The analytical sensitivity of P4 ELISA was 10 pg/mL. P4 levels in culture media were normalized to protein concentrations from corresponding cell lysates. For each treatment, normalized culture media P4 levels were expressed as relative values in comparison to the control treatment.

### Statistical analysis

The results are presented as the mean ± SEM of at least three independent experiments. All statistical analyses were analyzed by the PRISM software. Multiple comparisons were analyzed by one-way ANOVA, followed by Tukey’s multiple comparison tests. A significant difference was defined as *p* < 0.05. Values that are statistically different from one another (*p* < 0.05) are indicated by different letters. The values with any common letter are not significantly different.

## Results

### HB-EGF stimulates StAR expression in both KGN and hGL cells

**Fig. 1:**
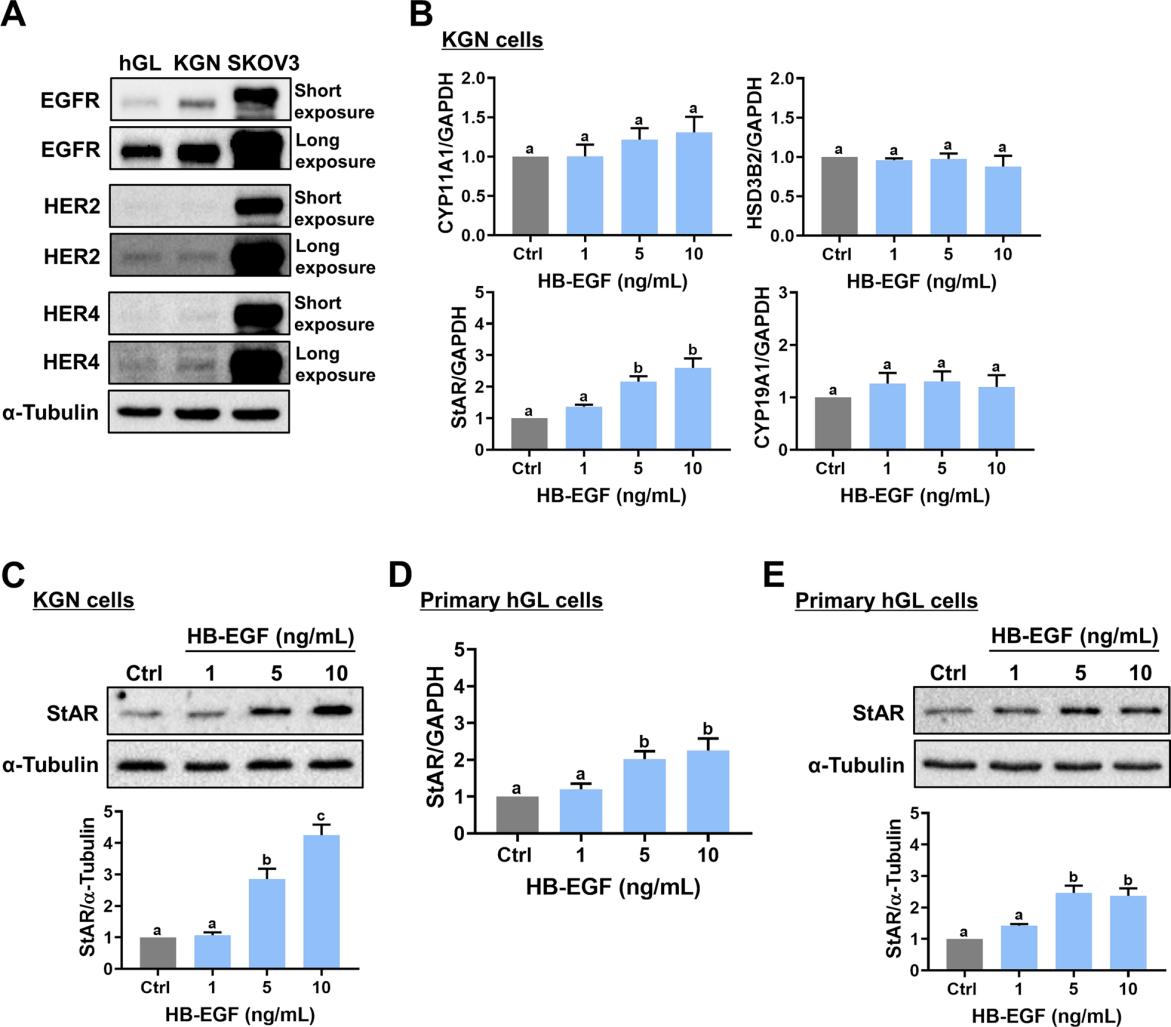
HB-EGF stimulates StAR expression in KGN and hGL cells.** A**, The protein levels of EGFR, HER2, and HER4 in KGN and hGL cells were examined by western blot. Human ovarian cancer cell line, SKOV3, was used as positive control.** B**, KGN cells were treated with 1, 5 or 10 ng/mL HB-EGF for 24 h. The mRNA levels of CYP11A1, HSD3B2, StAR, and CYP19A1 were examined by RT-qPCR.** C**, KGN cells were treated with 1, 5 or 10 ng/mL HB-EGF for 24 h. The StAR protein levels were examined by western blot.** D** and** E**, Primary hGL cells were treated with 1, 5 or 10 ng/mL HB-EGF for 24 h. The StAR mRNA levels (**D**) and protein levels (**E**) were examined by RT-qPCR and western blot, respectively. The results are expressed as the mean ± SEM of at least three independent experiments. Values that are statistically different from one another (*p* < 0.05) are indicated by different letters

Although no direct ligand has been identified, HER2/ErbB2 can be activated by dimerization with other ligand-bound ErbB receptors, which enhances their signaling [20, 21]. Since HB-EGF binds EGFR and HER4, we first examined the protein levels of EGFR, HER2, and HER4 in hGL and KGN cells. The human ovarian cancer cell line, SKOV3, which expresses high levels of EGFR, HER2, and HER4 [22], was used as a positive control for the expression of EGFR, HER2, and HER4. Consistent with previous findings, both hGL and KGN cells expressed EGFR, HER2, and HER4 [22]. The expression of EGFR was dominant compared to HER2 and HER4 (Fig. 1A). To examine the effect of HB-EGF on steroidogenesis, KGN cells were treated with 1, 5, and 10 ng/mL recombinant human HB-EGF for 24 h, and the mRNA levels of steroidogenesis-related genes were analyzed by RT-qPCR. As shown in Fig. 1B, none of any concentrations of HB-EGF significantly affected the mRNA levels of P450 side-chain cleavage enzyme (*CYP11A1*), 3β-Hydroxysteroid dehydrogenase (*HSD3B2*), and aromatase (*CYP19A1*). While treatment of 1 ng/mL HB-EGF had no significant effect, StAR mRNA levels were significantly upregulated by exposure to 5 or 10 ng/mL HB-EGF in KGN cells. A similar stimulatory effect of HB-EGF on StAR protein levels was observed by the western blot analysis (Fig. 1C). To further confirm the stimulatory effect of HB-EGF on StAR expression in human granulosa cells, primary hGL cells purified from follicular aspirates of IVF patients were used. As shown in Fig. 1D and E, treatment of primary hGL cells with HB-EGF upregulated both mRNA and protein levels of StAR. Therefore, 5 ng/mL HB-EGF was used for the following experiments.

### EGFR and HER4 mediate the stimulatory effect of HB-EGF on StAR expression

**Fig. 2:**
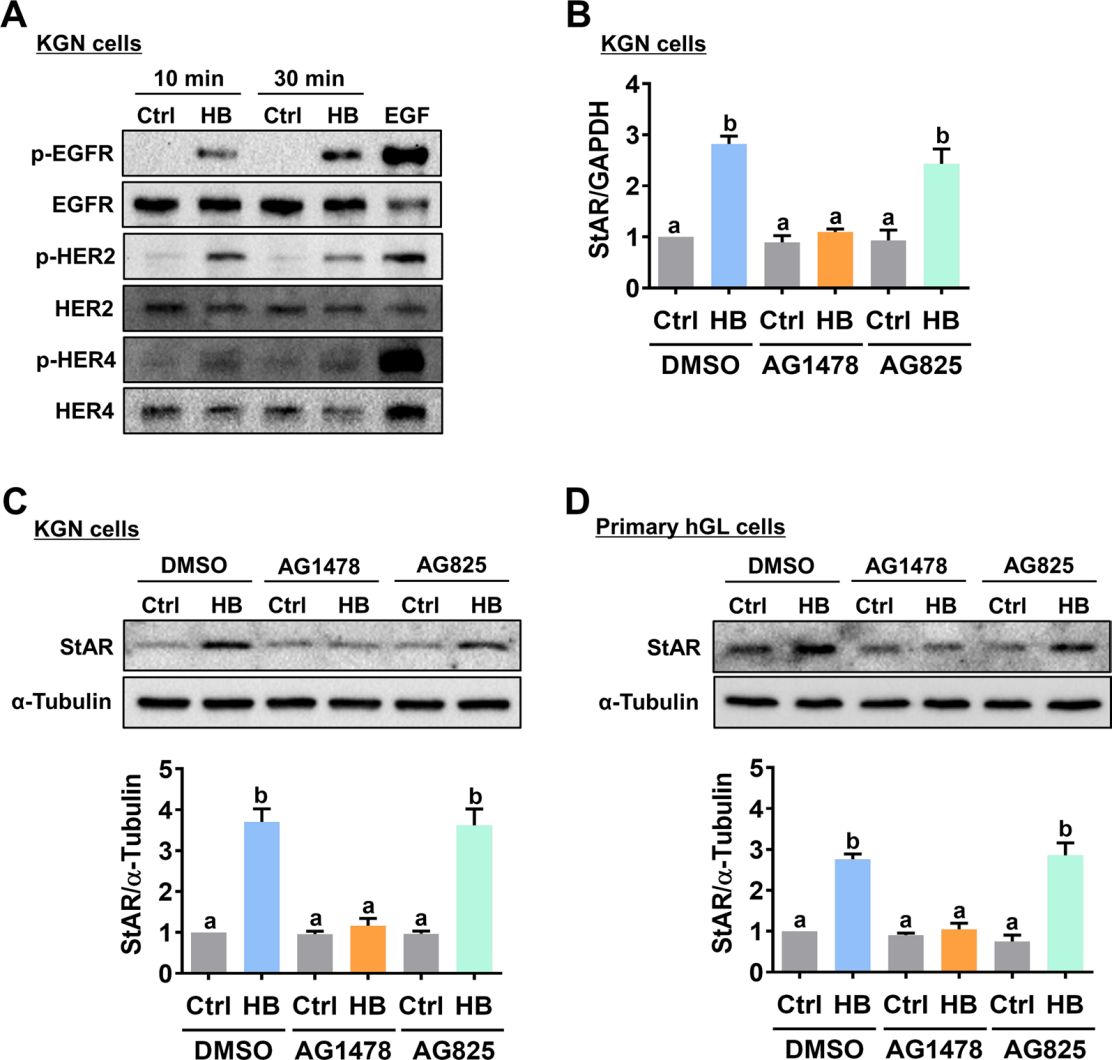
EGFR mediates HB-EGF-induced StAR expression. **A**, KGN cells were treated with 5 ng/mL HB-EGF (HB) for 10 and 30 min. The levels of phosphorylated and total forms of EGFR, HER2, and HER4 were determined by western blot. **B** and **C**, KGN cells were pretreated with vehicle control (DMSO), 10 µM AG1478 or 10 µM AG825 for 1 h, and then treated with 5 ng/mL HB-EGF (HB) for 24 h. The StAR mRNA levels (**B**) and protein levels (**C**) were examined by RT-qPCR and western blot, respectively. **D**, Primary hGL cells were pretreated with vehicle control (DMSO), 10 µM AG1478 or 10 µM AG825 for 1 h, and then treated with 5 ng/mL HB-EGF (HB) for 24 h. The StAR protein levels were examined by western blot. The results are expressed as the mean ± SEM of at least three independent experiments. Values that are statistically different from one another (*p* < 0.05) are indicated by different letters

**Fig. 3:**
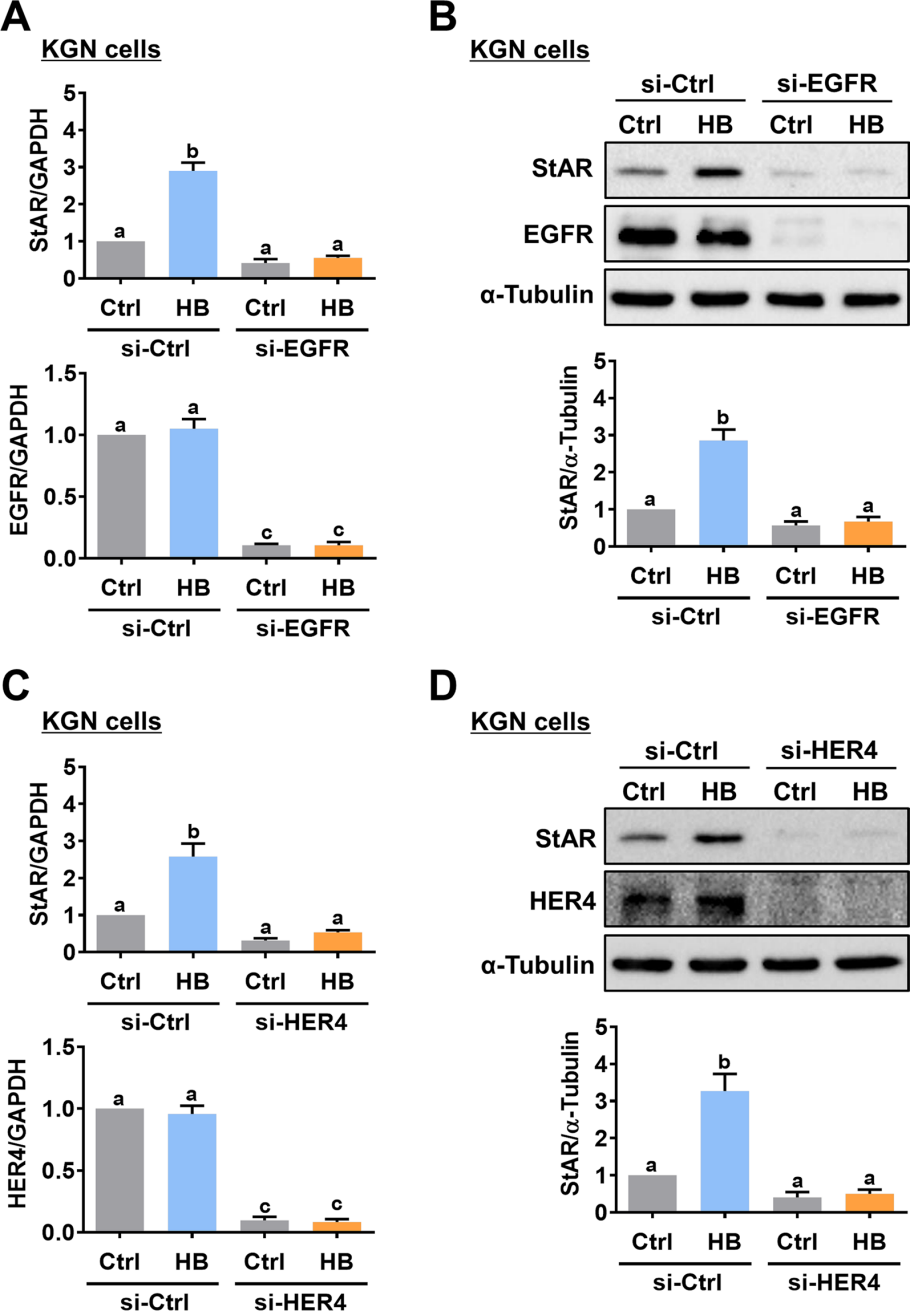
EGFR and HER4 are required for the HB-EGF-induced StAR expression. **A** and **B,** KGN cells were transfected with 50 nM control siRNA (si-Ctrl) or EGFR siRNA (si-EGFR) for 48 h, and then treated with 5 ng/mL HB-EGF (HB) for 24 h. The StAR and EGFR mRNA levels (**A**) and protein levels (**B**) were examined by RT-qPCR and western blot, respectively. **C** and **D**, KGN cells were transfected with 50 nM control siRNA (si-Ctrl) or HER4 siRNA (si-HER4) for 48 h, and then treated with 5 ng/mL HB-EGF (HB) for 24 h. The StAR and HER4 mRNA levels (**C**) and protein levels (**D**) were examined by RT-qPCR and western blot, respectively. The results are expressed as the mean ± SEM of at least three independent experiments. Values that are statistically different from one another (*p* < 0.05) are indicated by different letters

To examine whether HB-EGF activates EGFR, HER2, and HER4, KGN cells were treated with HB-EGF for 10 and 30 min. Western blot results showed that treatment of HB-EGF induced the phosphorylation levels of EGFR, HER2, and HER4, indicating the activation of these receptors (Fig. 2A). Cells treated with EGF for 10 min were used as positive controls. Next, we used pharmacological inhibitors to examine the requirement of EGFR and HER2 in HB-EGF-induced StAR expression. As shown in Fig. 2B, inhibition of EGFR by an EGFR tyrosine kinase inhibitor, AG1478, blocked the stimulatory effect of HB-EGF on the StAR mRNA levels. Interestingly, blocking HER2 function with the HER2 tyrosine kinase inhibitor, AG825, did not significantly affect the stimulatory effect of HB-EGF on the StAR mRNA levels. Western blot results confirmed the requirement of EGFR but not HER2 for the HB-EGF-induced StAR protein levels in both KGN and primary hGL cells (Fig. 2C and D). To directly examine the requirement of EGFR for HB-EGF-induced StAR expression, a siRNA-based gene silencing approach was applied to knockdown EGFR expression. Transfection of KGN cells with EGFR-specific siRNA downregulated endogenous mRNA and protein levels of EGFR. In addition, the knockdown of EGFR abolished the stimulatory effect of HB-EGF on the StAR mRNA and protein levels (Fig. 3 A and B). The same approach was used to examine the requirement of HER4 for HB-EGF-induced StAR expression. As shown in Fig. 3C and D, the HB-EGF-induced StAR mRNA and protein levels were blocked by the knockdown of HER4. Collectively, these results indicate that the stimulatory effect of HB-EGF on StAR expression in hGL cells is mediated by EGFR and HER4.

## ERK1/2 signaling mediates HB-EGF-induced StAR expression

**Fig. 4:**
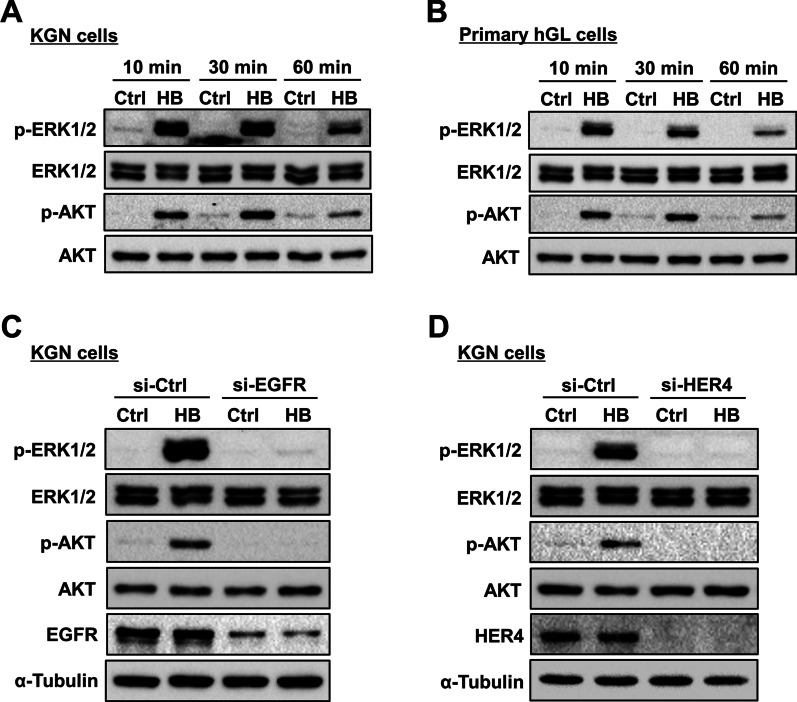
HB-EGF activates ERK1/2 and AKT signaling pathways in KGN and primary hGL cells. **A** and **B**, KGN (**A**) and primary hGL (**B**) cells were treated with 5 ng/mL HB-EGF (HB) for 10, 30, and 60 min. The levels of phosphorylated and total forms of ERK1/2 and AKT were determined by western blot. **C** and **D**, KGN cells were transfected with 50 nM control siRNA (si-Ctrl), EGFR siRNA (si-EGFR) (**C**), or HER4 siRNA (si-HER4) (**D**) for 48 h, and then treated with 5 ng/mL HB-EGF (HB) for 10 min. The levels of phosphorylated and total forms of ERK1/2 and AKT as well as the protein levels of EGFR or HER4 were determined by western blot

**Fig. 5:**
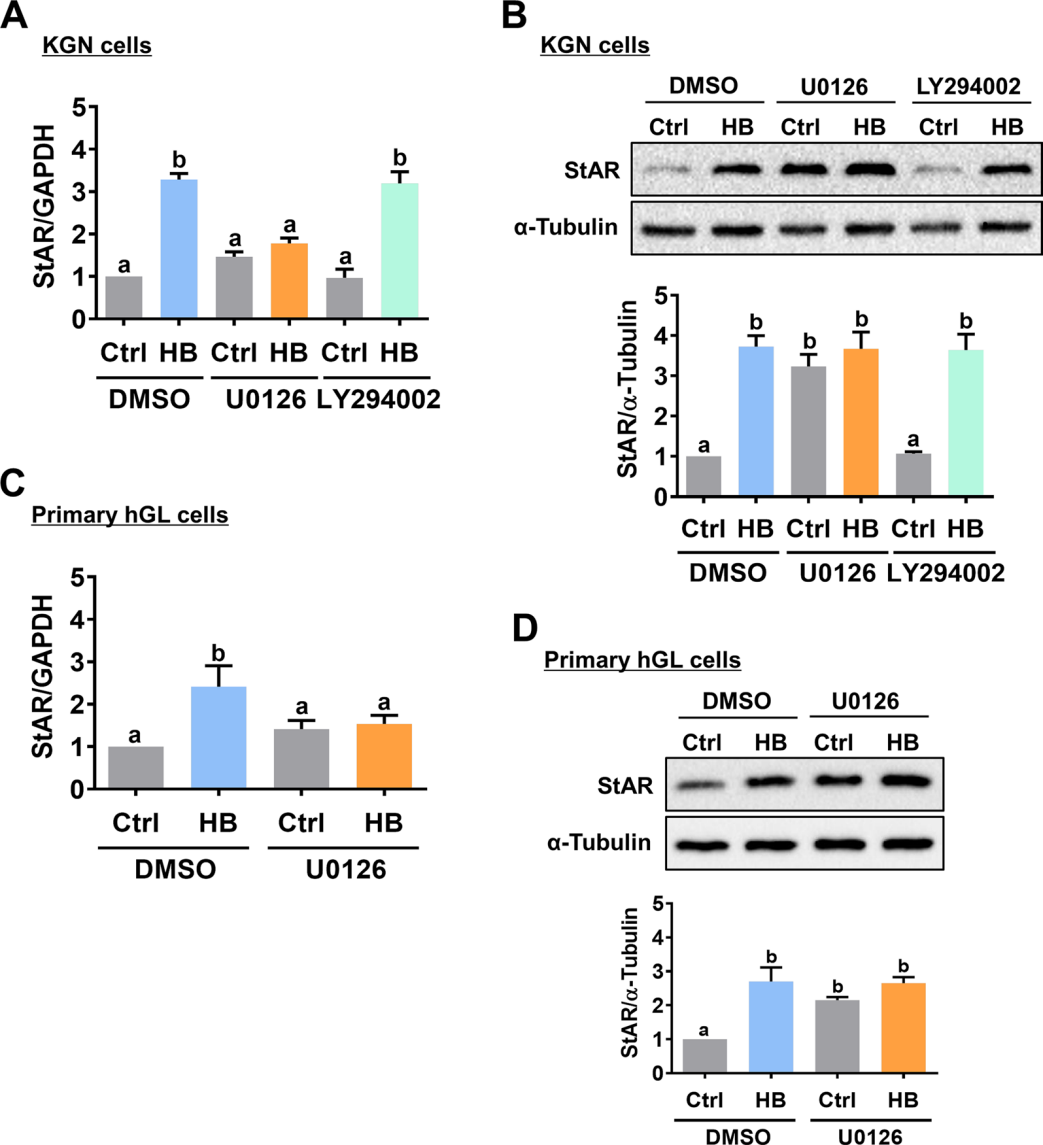
Activation of ERK1/2 signaling is required for the HB-EGF-induced StAR expression. **A** and **B,** KGN cells were pretreated with vehicle control (DMSO), 10 µM U0126 or 10 µM LY294002 for 1 h, and then treated with 5 ng/mL HB-EGF (HB) for 24 h. The StAR mRNA levels (**A**) and protein levels (**B**) were examined by RT-qPCR and western blot, respectively. **C** and **D**, Primary hGL cells were pretreated with vehicle control (DMSO) or 10 µM U0126 for 1 h, and then treated with 5 ng/mL HB-EGF (HB) for 24 h. The StAR mRNA levels (**C**) and protein levels (**D**) were examined by RT-qPCR and western blot, respectively. The results are expressed as the mean ± SEM of at least three independent experiments. Values that are statistically different from one another (*p* < 0.05) are indicated by different letters

Upon ligand binding, activated EGFR or HER4 transmits intracellular downstream signaling via the MEK/ERK1/2 and PI3K/AKT pathways [23]. Thus, we examined the effect of HB-EGF on the activity of these two signaling pathways in both KGN and hGL cells. As shown in Fig. 4A and B, HB-EGF treatment activated ERK1/2 and AKT in both KGN and hGL cells. In addition, the HB-EGF-activated ERK1/2 and AKT signaling pathways were blocked by the knockdown of EGFR or HER4 in KGN cells (Fig. 4C and D). Next, we tested a specific MEK inhibitor, U0126, and a PI3K inhibitor, LY294002, to further determine which signaling pathway is required for HB-EGF-induced upregulation of StAR expression. As shown in Fig. 5A, pretreatment with U0126 blocked the HB-EGF-induced upregulation of StAR mRNA levels. However, inhibition of the PI3K/AKT signaling pathway did not affect the stimulatory effect of HB-EGF on StAR mRNA levels. Western blot results showed that blocking the ERK1/2 signaling pathway with U0126 abolished the HB-EGF-induced upregulation of StAR protein levels in KGN cells (Fig. 5B). Similarly, inhibition of ERK1/2 signaling blocked the stimulatory effect of HB-EGF on the StAR mRNA and protein levels (Fig. 5C and D). Notably, treatment with U0126 increased the basal levels of StAR mRNA and protein levels. These results could be attributed to the off-target effect of the pharmacological inhibitor. Taken together, these results indicate that activation of the ERK1/2 signaling pathway is involved in HB-EGF-induced StAR expression in human granulosa cells.

### EGFR/HER4-mediated ERK1/2 activation is required for the HB-EGF-induced P4 production

**Fig. 6:**
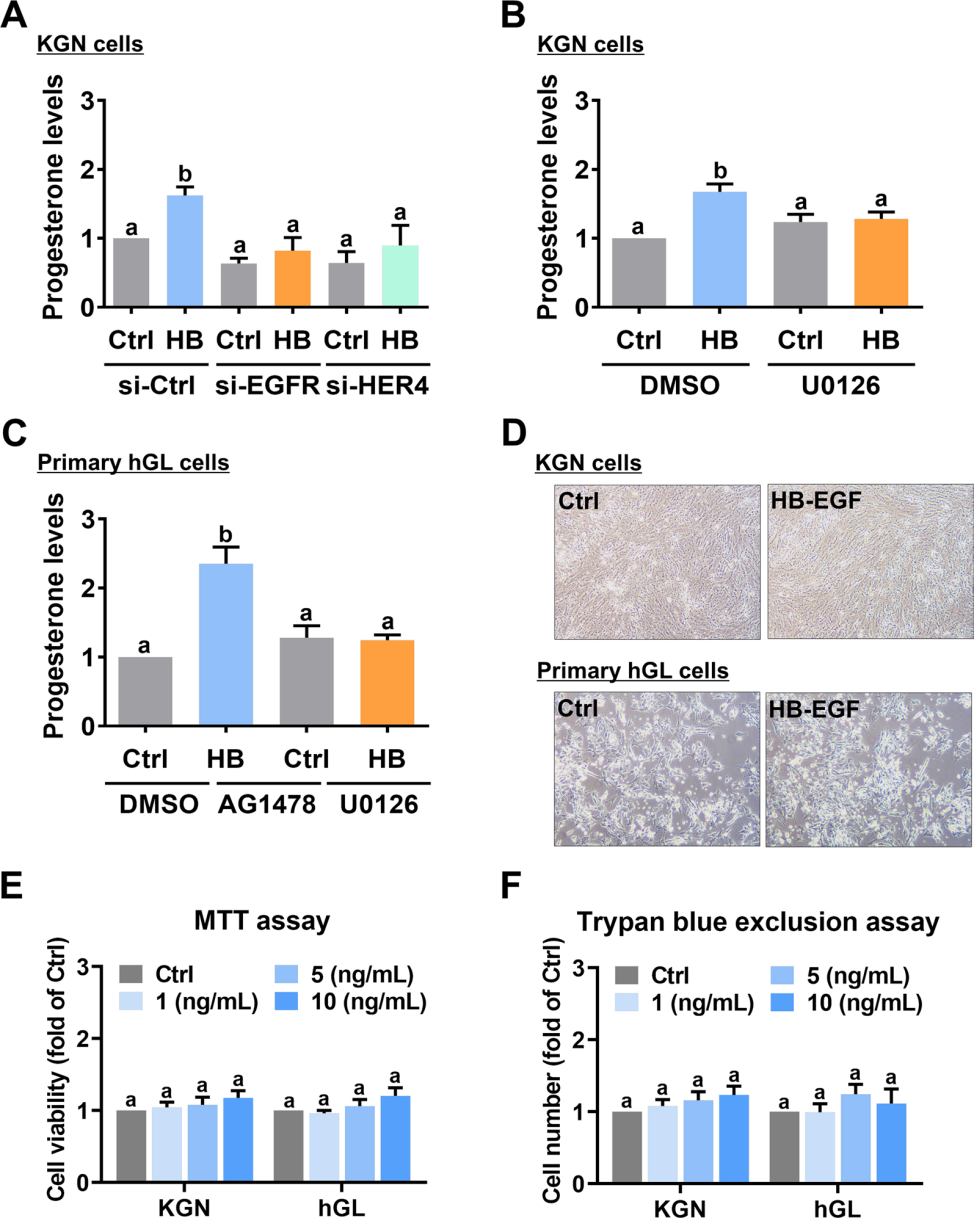
EGFR/HER4-activated ERK1/2 is required for HB-EGF-induced progesterone production. **A,** KGN cells were transfected with 50 nM control siRNA (si-Ctrl), EGFR siRNA (si-EGFR), or HER4 siRNA (si-HER4) for 48 h, and then treated with 5 ng/mL HB-EGF (HB) for 24 h. Progesterone (P4) levels in culture media were examined using ELISA. **B,** KGN cells were pretreated with vehicle control (DMSO) or 10 µM U0126 for 1 h, and then treated with 5 ng/mL HB-EGF (HB) for 24 h. Progesterone (P4) levels in culture media were examined using ELISA. **C,** Primary hGL cells were pretreated with vehicle control (DMSO) or 10 µM U0126 for 1 h, and then treated with 5 ng/mL HB-EGF (HB) for 24 h. Progesterone (P4) levels in culture media were examined using ELISA. **D,** KGN and primary hGL cells were treated with or without 5 ng/mL HB-EGF for 24 h. The resultant morphologies in KGN and primary hGL cells were microscopically examined. **E** and **F,** KGN and primary hGL cells were treated with 1, 5, or 10 ng/mL HB-EGF for 24 h. The cell viability was examined by MTT assay (**E**). The cell proliferation was examined by trypan blue exclusion assay (**F**). The results are expressed as the mean ± SEM of at least three independent experiments. Values that are statistically different from one another (*p* < 0.05) are indicated by different letters

Given the important role of StAR protein in the regulation of P4 production, we examined the effect of HB-EGF on the production of P4 in KGN cells as this cell line still preserves the function of steroidogenesis. ELISA results showed that KGN cells stimulated with HB-EGF produced P4 into the culture medium. This effect was blocked by the siRNA-mediated knockdown of EGFR or HER4 (Fig. 6A). In addition, HB-EGF-induced P4 production was blocked by the inhibition of the ERK1/2 signaling pathway (Fig. 6B). In primary hGL cells, we also observed the stimulatory effect of HB-EGF on P4 production. Similarly, the stimulatory effect of HB-EGF on P4 production was blocked by the inhibition of ERK1/2 signaling (Fig. 6C). Next, we examined the cell morphology after HB-EGF treatment and used MTT assay to determine whether the effects of HB-EGF on P4 production could be influenced by changes in cell phenotype or number. Treatment of KGN or primary hGL cells with HB-EGF did not affect the cell morphology (Fig. 6D). MTT assay showed that 24 h treatment of HB-EGF did not significantly influence the cell viability (Fig. 6E). MTT assay is a method to indirectly examine cell viability by measuring the mitochondrial metabolic rate [24]. It is possible that treatment of HB-EGF affected the mitochondrial metabolic activity of the cells, that led to considerable variation in results reported from MTT assays. Therefore, the effect of HB-EGF on cell proliferation was further confirmed by trypan blue exclusion assay. Similar to the results obtained from the MTT assay, treatment of HB-EGF for 24 h did not affect cell proliferation in both KGN and primary hGL cells, which indicates that the HB-EGF-increased P4 production was not attributed to the changes in cell number (Fig. 6F). Taken together, these results demonstrate that HB-EGF is a stimulator for P4 production in human granulosa cells, and this effect is mediated by the EGFR/HER-activated ERK1/2 signaling pathway.

## Discussion

The expression of HB-EGF has been examined in the ovary of different species. The expression pattern of ovarian HB-EGF is exhibited in a species-dependent manner. In rats, HB-EGF is expressed in luteal cells and granulosa cells of early follicles and all the developing follicles but not in preovulatory follicles [25]. HB-EGF is expressed predominantly in chicken oocytes. The expression of HB-EGF in chicken granulosa cells is very weak [26]. In pigs, HB-EGF is expressed in the corpus luteum. Its mRNA levels are highest on day 4 during luteal growth and development but start to decrease on day 10 when the steroidogenesis is very active [27]. In humans, HB-EGF and its receptors are expressed in the ovary [12]. Previous immunohistochemical and RT-qPCR results have demonstrated that HB-EGF expression is detected in granulosa-lutein cells in the early luteal phase. Its expression levels become most abundant in those cells in the mid-luteal phase, followed by a decline in the late luteal phase. Notably, HB-EGF is not expressed in preantral, antral, preovulatory follicles, or oocytes　[11]. These results suggest the role of HB-EGF in the regulation of human luteal function. The anti-apoptotic effect of HB-EGF has been reported in hGL cells [12]. While 24 h treatment of 10 nM HB-EGF (approx. 100 ng/mL) has no significant effect, KGN cell proliferation is slightly stimulated after 48 h of 10 nM HB-EGF treatment [28]. Our results showed that 24 h treatment with up to 10 ng/mL HB-EGF did not influence the KGN cell proliferation, which agrees with those previous findings. To the best of our knowledge, thus far, the effect of HB-EGF on steroidogenesis in the human corpus luteum is unclear. The present study provided evidence that HB-EGF treatment induced StAR expression in hGL cells and stimulated P4 production. The stimulatory effects of HB-EGF on StAR expression and P4 production were required EGFR/HER4-mediated ERK1/2 signaling pathway.

The follicular fluid contains a variety of molecules that can regulate ovarian function locally and thus provides an important microenvironment for the development of the ovarian follicle and the oocyte. The concentration of AREG in human follicular fluid is around 100 ng/mL and is the most abundant EGFR ligand. The follicular fluid AREG levels are 6,700 times and 23,000 times as high as those of TGF-α and EGF, respectively [29]. Two studies have measured the protein levels of HB-EGF in human follicular fluid, but the results are inconsistent. The protein levels of HB-EGF in follicular fluid are 350.42 pg/mL in IVF patients with successful implantation. In patients without implantation, the follicular fluid HB-EGF levels are 230 pg/mL [30]. However, a recent study shows that the follicular fluid HB-EGF levels in IVF patients without polycystic ovarian syndrome (PCOS) are around 3 ng/mL, which is 10-fold higher than the value reported by the study mentioned above [31]. Therefore, future studies will need to confirm the protein levels of HB-EGF in human follicular fluid. The mRNA levels of HB-EGF are downregulated in the granulosa cells of PCOS patients and in the ovaries of PCOS rats [32]. Our results showed that StAR expression was upregulated by HB-EGF in hGL cells. This finding, together with the downregulation of HB-EGF in PCOS ovaries, could explain the observation that StAR expression in hGL cells from PCOS patients is lower than that from non-PCOS patients [33].

Normal ovarian function can be regulated by a number of locally-produced hormonal factors that exert their effects in an autocrine and/or paracrine fashion. Our previous work has shown that the expression of StAR in hGL cells is downregulated by several members of the TGF-β superfamily that are known to be expressed in the human ovary [34–37]. Studies have made it clear that intracellular signaling pathways serve to regulate P4 production by regulating the expression of StAR [38]. We have shown that the siRNA-mediated knockdown of StAR decreases the basal and abolishes the melatonin-stimulated P4 production in hGL cells [39]. Therefore, understanding the regulation of StAR can increase our knowledge regarding the control of ovarian P4 production. It is known that EGF and AREG bind specifically to EGFR. In porcine granulosa cells, EGF alone does not affect the StAR mRNA levels, but it attenuates FSH-induced upregulation of StAR mRNA levels [40]. In mouse Leydig tumor cells, EGF stimulates StAR expression and P4 production [41]. Our previous study shows that activation of EGFR by AREG stimulates StAR expression and P4 production [17]. In this study, we showed that both EGFR and HER4 were activated by HB-EGF, and those were required for the HB-EGF-induced StAR expression and P4 production in KGN and hGL cells. The stimulatory effect of HB-EGF on StAR is also observed in PMSG-primed mouse granulosa cells [10]. Collectively, these results indicate that the effect of EGFR activation on StAR expression is in a species-dependent manner. Although HB-EGF can not activate HER2 directly, HER2 can be activated indirectly by heterodimerization with EGFR or HER4 [42]. Although HER2 was activated by HB-EGF in KGN cells, using HER2 inhibitor, we revealed that HER2 was not required for the HB-EGF-induced StAR expression. In human cytotrophoblast cells, HB-EGF induces an integrin switching from α6β4 to α1β1. Similar to our findings, blocking the HER2 activity with AG825 does not affect the effect of HB-EGF on integrin switching [43]. The ERK1/2-CREB pathway has previously been implicated in mediating StAR expression and P4 production. In granulosa cell-specific *Erk1/2* knockout mice (*Erk1/2*^*gc−/−*^), hCG-induced *Star* gene expression in granulosa cells is impaired [44]. CREB is involved in epigallocatechin-3-gallate-stimulated StAR expression in KGN cells [45]. Our results showed that the activation of ERK1/2 but not AKT signaling was required for the HB-EGF-induced StAR expression and P4 production. Whether CREB is activated by HB-EGF and required for HB-EGF-induced StAR expression and P4 production remains unknown and warrants further investigations.

## Conclusion

In summary, the present study, for the first time, reports that the StAR expression and P4 production in hGL cells can be induced by the HB-EGF treatment. The stimulatory effects of HB-EGF on StAR expression and P4 production are mediated by EGFR and HER4. In addition, the activation of ERK1/2 is required for HB-EGF-induced StAR expression and P4 production. This study increases the understanding of the biological function of HB-EGF and provides a novel mechanism for the regulation of ovarian steroidogenesis, which could help to develop therapeutic methods for female ovarian diseases.

## Data Availability

The data that support the findings of this study are available from the corresponding author upon reasonable request.

## Abbreviations

AREG: Amphiregulin
EGF: Epidermal growth factor
FSH: Follicle-stimulating hormone
HB-EGF: Heparin-binding epidermal growth factor-like growth factor
hGL: Human granulosa-lutein cells
IVF: In vitro fertilization
LH: Luteinizing hormone
PCOS: Polycystic ovarian syndrome
P4: Progesterone
StAR: Steroidogenic acute regulatory protein
TBS: Tris-buffered saline
